# Effects of community peer-led programmes for people with spinal cord injury in Sweden–the INTERnational project for the evaluation of active rehabilitation (INTER-PEER)

**DOI:** 10.1038/s41393-025-01119-4

**Published:** 2025-10-06

**Authors:** Anestis Divanoglou, Erik Berndtsson, Tomasz Tasiemski, Carolina Saskia Fellinghauer, Sophie Jörgensen

**Affiliations:** 1 Clinical Department of Rehabilitation Medicine in Linköping, Linköping University Hospinal, Region Östergötland, Linköping, Sweden; 2 Department of Health, Medicine, and Caring Sciences, Linköping University, Linköping, Sweden; 3 RG Active Rehabilitation, Solna, Sweden; 4 Department of Adapted Physical Activity, Faculty of Sport Sciences, Poznan University of Physical Education, Poznan, Poland; 5 Swiss Paraplegic Research, Nottwil, Switzerland; 6 Department of Health Sciences, Research Group Rehabilitation Medicine, Lund University, Lund, Sweden; 7 Department of Rehabilitation Medicine, Skåne University Hospital, Lund, Sweden

**Keywords:** Rehabilitation, Patient education

## Abstract

**Study design:**

Longitudinal prospective cohort study.

**Objectives:**

To assess the effects of Active Rehabilitation (AR) training programmes for individuals with spinal cord injury (SCI) on physical independence, self-efficacy and wheelchair skills, and to identify factors that are associated with gains in these outcomes.

**Setting:**

Eight consecutive AR programmes in Sweden.

**Methods:**

Participants (n = 111) with traumatic or nontraumatic SCI, aged 16 years and older, were evaluated at the start (T1) and completion (T2) of the programme, and at 3-month follow-up (T3). Assessments included standardised self-reported outcome measures (T1-T2-T3) and a practical wheelchair skills test (T1-T2).

**Results:**

After attending the short, intensive peer-led AR programmes, participants reported gains in physical independence, especially in dressing and washing, bowel management, bed mobility, and transfers. Wheelchair skills improved, while improvements in aspects of self-efficacy and resilience were observed only at programme completion. Gains in physical independence and self-reported wheelchair skills were present at the 3-month follow-up. With few exceptions, examined predictors did not explain the observed outcome gains.

**Conclusion:**

AR programmes offer an effective, low-cost opportunity to improve essential and challenging aspects of physical independence and wheelchair skills among community-dwelling individuals with SCI. Immediate gains in aspects of self-efficacy and resilience–though not sustained at follow-up–may provide an initial momentum for future behavioural change, particularly among individuals who struggle to adjust to life after SCI. These findings strongly support the inclusion of intensive, residential, community-based peer-led programmes as a key component of the rehabilitation continuum for people with SCI.

## Introduction

Even in countries with comprehensive systems of care, transition from in-patient rehabilitation to community remains a considerable challenge for many individuals with a new spinal cord injury (SCI) [1, 2]. To improve transition to home and to achieve a higher degree of participation, more focus needs to be given to community rehabilitation programmes [3]. Peer support services have been addressed by international recommendations [4, 5] and scientific publications [6–8] as an important complement to clinical rehabilitation services [9]. Peer support was an expressed service need among approximately one in three community dwelling individuals with SCI [10, 11], and was found to be the least fulfilled among a list of 20 service needs [10].

There are various models of SCI peer mentorship internationally [12]. One distinct approach is Active Rehabilitation (AR), which we have previously described using the Template for Intervention Description and Replication (TiDieR) guidelines [13]. AR consists of peer-led (delivered by trained peer mentors with lived experience of SCI), time-limited (typically 7–10 days), residential training programmes. These programmes take place in community settings (e.g., sports centres with accommodation facilities) and comprise an intensive schedule of one-to-one and group-based training in activities of daily living (ADL), athletic and recreational activities, educational sessions, and informal peer interactions [13].

Originally developed in Sweden in the 1970s, the AR concept has since been adapted and implemented in over 20 countries–including high-, middle- and low-income–under various names [14]. The international development has been driven by organisations such as RG Aktiv Rehabilitering (Sweden), FAR (Poland) and Motivation (UK) [14], as well as by individuals, including co-author Tomasz Tasiemski. In 2025, the Active Rehabilitation International (ARI) network was launched to further support the standardisation and global development of the AR model. In Sweden as of 2022, the full cost for mentees to participate in AR programmes is approximately 3700 SEK/day (~320 Euro/day; personal communication with head of RG AR in Sweden), largely subsidised through government funding and sponsorships [14]. Despite anecdotal evidence about positive effects of AR training programmes, there is little scientific evidence available [15, 16].

It has been argued that peer mentors are an important resource, because they have a high level of relatedness with mentees and they constitute a living example of what mentees could achieve [17]. Interacting with peer mentors is essential to building courage, evaluating the sense of the possible and creating a new image of the self [18]. A study on the effects of the inaugural AR programme in Botswana showed that AR can play an important role in promoting physical independence, wheelchair mobility and injury-management self-efficacy in community-dwelling individuals with SCI [15]. We have also found that AR programmes in Poland improve wheelchair skills with improvements largely retained at the 3-month follow-up [16]. AR programmes can also have positive effects on peer mentors by promoting their own growth (challenging themselves, boost of motivation, setting new personal goals) [19]. There is also rich anecdotal evidence that AR programmes can be positive life-changing experiences. Despite such reports, there is still a scarcity of comprehensive and convincing evidence about the effects of AR programmes on mentees with SCI. In the Swedish context, where the concept of AR was originally developed [13], evidence is completely lacking. Moreover, given the demographic shifts in traumatic SCI in Nordic countries during the last 20 years [20], it is important to explore whether AR programmes adequately address the needs of emerging sub-groups, including individuals with incomplete lesions, older adults, and women.

The current study forms part of the International Project for the Evaluation of Active Rehabilitation (INTER-PEER), which is the first comprehensive scientific evaluation of the AR training programmes for mentees with SCI [13] in the Swedish context. The primary objective of this study was to assess the effects of AR training programmes on physical independence, self-efficacy and wheelchair skills, and to identify demographic and injury characteristics that are associated with potential gains in these outcomes. The secondary objective was to assess the effects on community participation, life satisfaction and resilience.

## Methods

### Design

This prospective cohort study is based on the INTER-PEER protocol [13] and comprises the first systematic and comprehensive evaluation of the effects of AR training programmes among individuals with SCI in Sweden. The reporting for this study follows the Strengthening the Reporting of Observational studies in Epidemiology (STROBE) statement for cohort studies [21].

### Participants

Between April 2018 to April 2022, all consecutive participants in Swedish AR programmes that last for at least 7 days were invited to participate in the INTER-PEER given they met the following inclusion criteria: (1) existing SCI (acquired traumatic and non-traumatic, and congenital, e.g., spina bifida); (2) 16 years of age or older; (3) able to comprehend and answer written questions. Overall, data from eight AR programmes were collected: three in 2018, three in 2019, one in 2021 and one in 2022 (digital programmes in 2020–2021 due to Covid-19 pandemic were not included).

### Data collection

Participant evaluation took place at 3 time points: at the commencement (T1, baseline) and completion of the training programme (T2), and 3 months after the end of the AR programme (T3). At T1 and T2, data were collected using digital tablet devices on which participants completed the online survey at the platform Survey Monkey (www.surveymonkey.com/) and through a practical wheelchair skills assessment administered by peer mentors. At the end of each programme, the on-site data collection coordinator completed a form to monitor how well each programme met the INTER-PEER fidelity criteria. At T3, the participants were provided with an individualised link to complete the survey through their own device in their own time.

Data on sociodemographic and injury-related factors were collected using 17 questions adapted from the International Spinal Cord Injury Community Survey [22].

### Outcome measures

The focus and content of the AR programme informed the decision about primary and secondary outcome measures [13]. The published INTER-PEER protocol provides more details about each of these outcome measures [13]. The primary outcome measures evaluated SCI-specific physical independence through the Spinal Cord Independence Measure Self-report (SCIM-SR) comprising self-care, respiration and sphincter management, mobility in room and toilet, and mobility indoors and outdoors domains; self-efficacy through the Moorong Self-efficacy scale (MSES) further distinguishing into personal (disability management) self-efficacy, social and general self-efficacy; practical wheelchair skills measured using the Queensland Evaluation of Wheelchair Skills (QEWS) test; self-reported wheelchair skills using the Wheelchair Skills Test questionnaire (WST-Q) which looks into capacity and confidence; resilience through the Connor-Davidson Resilience scale (CD-RISC); community participation through the Utrecht Scale for Evaluation of Rehabilitation-Participation (USER-P) comprising frequency and restrictions domains; and life satisfaction through the generic Life Satisfaction scale (LiSat-11). As part of the INTER-PEER, we translated SCIM-SR and MSES into Swedish and tested the psychometric properties of the translated versions. All chosen outcome measures have good psychometric properties [23, 24].

### Statistical analyses

Descriptive statistics, i.e., mean and standard deviation (SD), frequency (n), percent (%), median and interquartile range (IQR) were used to present demographic and injury characteristics. As most instruments have score ranges between 0–100 and comprise a substantial number of items, ordinal scores were treated as interval-scaled [25].

We analysed and reported outcomes at two levels:

- *Instrument level analysis shows the mean and 95% confidence intervals of the score distributions of the respective outcome measures*. The mean difference between measuring points (T1 and T2; T1 and T3) were calculated to show the magnitude of change over time. Changes in total and domain scores across the three measurement time points were analysed with a mixed model analysis of variance (ANOVA) for repeated measures with auto-regressive covariance structure. Pairwise comparisons of total and domain scores used the paired t-test and its effect sizes (d), i.e. a standardised mean difference. Using Cohen’s criteria, an effect size ≥0.20 and <0.50 was considered small, ≥0.50 and <0.80 medium and ≥0.80 large [26].
- *At an individual item level*, we used the Wilcoxon signed-rank test to identify score changes between respective time points. In the Results, we report the items in which participants improved. In Supplementary Tables we report the number of individuals who improved in the respective items, as well as the proportion of those who improved among those below the highest possible score at T1 (i.e., those with room for improvement). In line with previously published studies, data for individual items of LiSat-11 were dichotomized into not-satisfied (scores 1–4) and satisfied (scores 5–6) [27], data for USER-P restrictions were dichotomized into restricted (0–2) and not restricted (3) [28], with differences between time points then analysed using the McNemar test.
  Further, we identified individuals who reached a small clinically important difference. Thresholds for small clinically important differences were determined as significant if the change was larger than SDstart x 0.2 [29, 30]. We report the number (% of valid cases) reaching clinically important differences for domain and total scores for all primary outcome measures (i.e. SCIM-SR, MSES, WST and QEWS). All cut-offs are provided in Supplementary Table 1.

The changes in domain and total scores of SCIM, MSES, QEWS, and WST between T2-T1/T3-T1 (dependent variables) were modelled with univariable regression analyses to investigate their association with each sociodemographic (i.e., sex, age), time since injury, level and completeness of injury (i.e., complete paraplegia, incomplete paraplegia, complete tetraplegia, incomplete tetraplegia), cause of injury (traumatic, non-traumatic), and the number of previously attended programmes coded as new mentee (first or second programme) or recurrent mentee (attended more than two programmes). These independent variables were chosen based on the objectives of the study, previous research and experiences from the AR programmes. Independent variables with a p-value below 0.2 [31] were then included in multivariable linear regression models, to predict both domain and total score change of SCIM, MSES, QEWS, and WST. Adjusted R [2] was used as a measure of explained variance. Influential cases were determined using Cook’s distance and the values of the standardised residuals. Values of Cook’s distance above 1 signalled the presence of influential cases. Analysis with Variance Inflation Factor (VIF) and tolerance give the amount of multicollinearity among the variables, and values of above 10 or below 0.2, respectively, are cause for concern [32]. Further, residuals were tested for heteroscedasticity and normal distribution [32]. A few models had 1–3 cases with standardised residuals larger than 3. Removing these cases did not change the inferences and the results are presented with all cases included.

The marginal means report the estimated change from the mean in the outcome for each category level of the independent variables, adjusted for the other independent variables in the model. Marginal means support the interpretation and understanding of the effects found in the regression analyses. All statistical analyses were performed using the Statistical Package for the Social Sciences (SPSS) version 28. The level of significance used was p < 0.05.

## Results

### Fidelity Criteria

On average there were 14 participants with SCI and 11 peer mentors with SCI and, in each of the eight included AR training programmes, 1–2 mentees per peer mentor. Peer mentors led between 96–100% of all scheduled sessions; on average there were 277 min of scheduled sessions per day comprising 101 min of activities of daily living (ADL) and wheelchair skills training; 109 min dedicated to physical training, sports and recreational activities; 68 min dedicated to formal educational sessions. Programmes lasted between 7–11 days (on average 8 days). Supplementary Table 2 provides further details for all INTER-PEER fidelity criteria.

### Mentee characteristics and reported complications

**Fig. 1:**
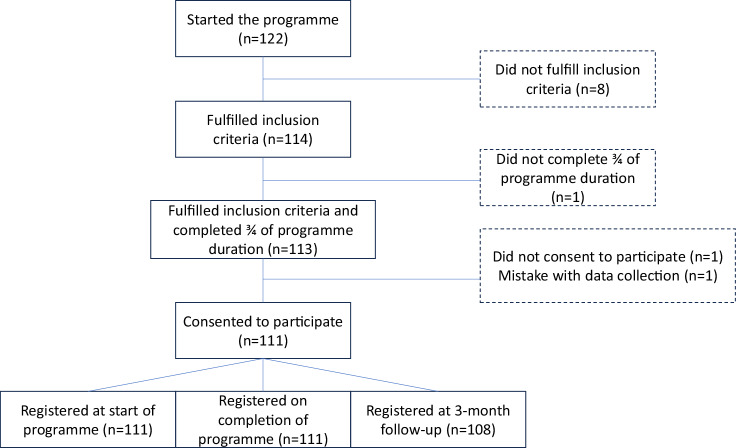
Flowchart for the inclusion and exclusion of study participants.

Figure 1 provides a flowchart for the inclusion and exclusion of participants. Out of a total of 122 participants in the 8 consecutive AR programmes, 111 participants (87 unique individuals, as 24 individuals attended multiple programmes within the study) were included in the study (Table 1), with 62% being first comers. In total, 65% were males, median (IQR) age was 43 (26) years, and the participants had been living with SCI on average for 1 (2) year. 85% had a traumatic lesion, 49% had tetraplegia and 44% had a complete lesion. 35% had at least a university degree, 35% were working and 19% were studying at the time of the programme. Supplementary Table 3 shows the reported complications prior, during and after the training programme. Uncomplicated falls was by far the most reported complication during the programme (28%). Out of the 111 participants, one reported a fall resulting in an injury during the programme, and 5 reported a skin injury due to fall or other activity.

**Table 1:** Demographic and injury characteristics of the 111 study mentees with spinal cord injury (SCI).

*Demographics and injury characteristics*	*Mentees with SCI (n* = *111)*
*Sex (n, %)*	
Male	72 (64.9%)
Female	39 (35.1%)
*Age (median years; IQR)*	43; 26
16–30	32 (28.8%)
31–45	25 (22.5%)
46–60	40 (36.0%)
61–75	13 (11.7%)
76+	1 (0.9%)
*Marital status (n, %)*	
Single	38 (34.2%)
Married	36 (32.4%)
Cohabiting or in a partnership	26 (23.4%)
Separated or divorced	9 (8.1%)
Widowed	2 (1.8%)
*Education (n, %)*	
Basic (1–9 years)	10 (9.0%)
Secondary (10–12 years)	39 (35.1%)
Post-secondary	23 (20.7%)
Bachelor	14 (12.6%)
Post-graduate	25 (22.5%)
*Employment status (n, %)*	
Employed	39 (35.1%)
Employed, currently on sick leave^a^	16 (14.4%)
Unemployed	5 (4.5%)
Student	21 (18.9%)
Retired due to health condition	32 (28.8%)
Retired due to age	6 (5.4%)
Missing	11 (9.9%)
*Monthly household income (n, %)*	
<20,000 SEK^b^	32 (29.6%)
20,000–30,999 SEK^c^	22 (20.4%)
31,000–42,999 SEK^d^	26 (24.0%)
≥43,000^e^	28 (16.0%)
*Level and completion of SCI (n, %)*	
Complete Paraplegia	24 (21.6%)
Incomplete Paraplegia	33 (29.7%)
Complete Tetraplegia	24 (21.6%)
Incomplete Tetraplegia	29 (26.1%)
Missing	1 (0.9%)
*Cause of traumatic SCI (n, %)*	92 (85.2%)
Sport	19 (20.7%)
Recreation	20 (21.7%)
Work related	8 (8.7%)
Traffic accident	24 (26.1%)
Assault	4 (4.3%)
Fall <1 m	3 (3.3%)
Fall >1 m	12 (13.0%)
Other	2 (2.2%)
*Cause of non-traumatic SCI (n, % of NTSCI)*	16 (14.8%)
Tumour benign	1 (7.7%)
Vascular disorders	4 (30.8%)
Infection	2 (15.4%)
Other	6 (46.2%)
Missing	3 (18.8%)
*Time since injury (median years; IQR)*	1; 2
0–6 months	13 (11.7%)
7–12 months	25 (22.5%)
13–24 months	28 (25.2%)
25–60 months	31 (27.9%)
61+ months	14 (12.6%)
*Previously attended programmes (n, %)*	
New mentees (0–1 previous *programmes*)	69 (62.2%)
Recurrent mentees (>1 previous *programmes*)	42 (37.8%)
*Main mode of mobility*	
Manual wheelchair	93 (83.8%)
Power-driven wheelchair	1 (0.9%)
Walking aid	6 (5.4%)
No mobility aid	4 (3.6%)
Missing	7 (6.3%)

^a^sick leave for more than 3 months.

^b^<1750 EUR.

^c^1750 - 2699 EUR.

^d^2700 - 3749 EUR.

^e^≥3751 (date of conversion rate 17.10.2024).

### Physical independence

For total SCIM-SR score, there were large gains at programme completion that decreased to small effect size gains at the 3-month follow-up (Table 2). A total of 39% achieved a small clinically meaningful improvement (≥4 points) at completion, with 34% achieving that at follow-up.

**Table 2:** Effects of AR training program on SCIM-SR, MSES, WST, CD-RISC (measured at T1: baseline; T2: completion of programme; T3: 3 months follow-up).

	*T1*		*Difference T2-T1*				*Difference T3-T1*			
	*n*	*Mean (95% CI)*	*n*	*Mean difference (95% CI)*	*Effect size (d)*	*Clinically important difference*–*small (%)*^*b*^	*n*	*Mean difference (95% CI)*	*Effect size (d)*	*Clinically important difference–small (%)*^*b*^
SCIM Total score (0-100)^a^	*109*	60.2 (56.8–63.5)	*108*	2.9 (1.6–4.1)**	0.8	39%	*103*	2.2 (0.4–4.0)**	0.4	34%
Self-care (0-20)^a^		15.4 (14.5–16.3)		1.2 (0.8–1.7)**	0.9	47%		0.6 (-0.1–1.2)		42%
Respiration and sphincter (0-40)^a^		26.5 (25.1–27.9)		0.8 (-0.1–1.7)		34%		0.9 (-0.4–2.1)		31%
Mobility in room and toilet (0-10)^a^		7.6 (7.1–8.1)		0.3 (0.0–0.6)*	0.4	30%		0.6 (0.1–1.0)**	0.5	37%
Mobility indoors and outdoors on even surfaces (0-30)^a^		10.6 (9.3–11.9)		0.3 (-0.3–0.9)		18%		0.2 (-0.6–1.0)		21%
Moorong Self-efficacy Scale (16-112)^a^	*111*	5.4 (5.2–5.5)	*110*	0.3 (0.2–0.4)**	0.7	51%	*106*	0.1 (-0.1–-0.3)		40%
Social function^a^		5.8 (5.6–6.0)	*110*	0.2 (0.1–0.4)**	0.6	47%		0.0 (-0.2–0.2)		41%
General self-efficacy^a^		5.3 (5.1–5.6)	*110*	0.2 (0.1–0.4)**	0.3	52%		0.1 (-0.2–0.3)		54%
Personal function^a^		5.3 (5.0–5.5)	*110*	0.3 (0.1–0.5)**	0.6	37%		0.2 (-0.1–0.4)		40%
WST-Q Capacity (0-100)^a^	*102*	65.4 (61.0–69.8)	*104*	8.2 (5.8–10.6)**	1.2	59%	*88*	7.1 (3.7–10.6)**	0.7	59%
WST-Q Confidence (0-100)^a^	*95*	57.5 (52.7–62.3)	*99*	8.4 (5.0–11.9)**	0.8	65%	*78*	7.3 (2.3 -12.2)**	0.5	61%
CD-RISC (0-100)^a^	*74*	69.5 (65.4–73.7)	*73*	5.1 (2.4–7.7)**	0.7		*68*	2.0 (-5.7–1.7)		

*p < .05. **p < .01.

^a^Mixed model ANOVA for repeated measures

^b^% is based on individuals reaching small clinically important difference out of those who have potential for improvement (cases starting with maximum points are excluded from this calculation). Clinically important difference was calculated for primary outcome measures only.

*SCIM-SR* Spinal Cord Independence Measure Self-Report, *MSES* Moorong Self-Efficacy Scale, *WST-Q* Wheelchair Skills Test Questionnaire, *CD-RISC* Connor–Davidson Resilience Scale.

Self-care was the domain with the largest effects in SCIM-SR at programme completion (d = 0.9), where 47% reached a small clinically meaningful improvement (≥1 point). At the 3-month follow-up, domain score effects were not retained at a group level, but 42% reached a small clinically meaningful improvement. At an individual item level (Supplementary Table 4), there were improvements in dressing upper and lower limbs, in grooming, washing upper limbs (UL) and lower limbs (LL) at programme completion and at follow-up.

In the respiratory and sphincter management domain, 34% reached a small clinically meaningful improvement (≥1 point) at completion and 31% at follow-up, but there were no significant changes at group level. Toilet use was the item with the highest proportion of participants (more than 90%) who had not reached the highest possible score at programme start. One in five of those participants reported gains at completion, but not at follow-up. There were also improvements in bowel management at follow-up.

In the mobility in room and toilet domain, small effects were reported at programme completion (d = 0.4), which were further increased to moderate effects (d = 0.5) at the 3-month follow-up. Thirty percent reached at least a small clinically important difference (≥1 point) at programme completion and 37% at follow-up. There were improvements in wheelchair to toilet transfer at completion and follow-up, and in transfer from wheelchair to bed at completion.

In mobility indoors and outdoors, there were no significant changes in domain scores at group level. Approximately 20% reached at least a small clinically important difference (≥1 point) at programme completion and at follow-up. There were improvements in transfer from wheelchair to car at completion, but not at follow-up, and in floor to wheelchair transfer at programme completion and follow-up.

### Self-efficacy

For the total MSES score, moderate effect size gains (d = 0.7) were achieved at programme completion, which were not retained at 3-month follow-up (Table 2). A total of 51% achieved a small clinically meaningful improvement (≥0.2 points) at completion, and 40% at follow-up.

For the dimension of personal (disability management) self-efficacy, there were moderate gains at completion which were not maintained until follow-up. A small clinically meaningful improvement was reached by 37 and 40% at completion and follow-up, respectively. The largest improvements were found in relation to avoiding bowel accidents and being an active member of the household (Supplementary Table 5). Confidence with avoiding bowel accidents was the single item in MSES where gains were sustained at the 3-month follow-up.

For Social self-efficacy, there were moderate gains at completion which were not maintained until follow-up. A small clinically meaningful improvement was reached by 47 and 41% at completion and follow-up, respectively. The largest improvements were found in relation to finding hobbies and leisure and maintaining contact with people. For General self-efficacy, there were small gains at completion but not at follow-up. A small clinically meaningful improvement in General self-efficacy was reached by about 50% at completion and follow-up. The largest improvements were found in relation to dealing with unexpected problems.

### Wheelchair skills

The participants achieved improvements of small effect size in their wheelchair skills assessed with QEWS (d = 0.2) at completion (Table 3). In total, 41% reached a small clinically meaningful improvement (≥1 point). These gains were related to ascending and descending a gutter, maintaining balance on the back wheels and the six-minute push test (Table 3). No improvements were found in negotiating an indoor circuit or ascending and descending a ramp, where participants were already at a high level.

**Table 3:** Effects on Wheelchair Skills test (measured at T1: baseline and T2: completion of programme).

	*Baseline*	*Completion of programme*		
QEWS items	*Mean (SD)*	*Mean (SD)*	*P-value*	*Effect size (d)*
1. Negotiating an indoor circuit^a^	5.0 ± 0.1	5.0 ± 0.1	0.564	
2. Ascending and descending a ramp^a^	4.1 ± 1.1	4.2 ± 1.3	0.439	
3. Maintaining balance on back wheels^a^	3.3 ± 2.1	3.5 ± 2.0	0.027	0.1
4. Ascending and descending a gutter^a^	1.7 ± 1.6	2.2 ± 1.6	<0.001	0.3
5. Six-minute push test^a^	3.2 ± 1.3	3.3 ± 1.4	0.043	0.1
Total score^b^	17.5 ± 5.0	18.3 ± 5.2	<0.001	0.2

^a^Wilcoxon Signed Rank test.

^b^Paired t-test.

In terms of wheelchair skills assessed with the WST-Questionnaire, participants achieved large gains at programme completion, and moderate gains at follow-up (Table 2; Supplementary Table 6). In total, 59% reached at least a small clinically meaningful improvement (≥5 points) at completion and follow-up in WST-capacity. Individual skills that most participants could not master in the beginning of the programme involved managing curbs, steep inclines and performing a wheelie, i.e., balancing the wheelchair on the rear wheels (items 18, 26, 27, 30, 31). More than a third of participants not mastering these skills at the start of AR programme achieved gains at completion and at the 3-month follow-up.

WST-Q confidence improved from programme start to completion with medium to large effect size gains at both total and domain score levels. For many skills, the success rate remained the same between completion and follow-up (e.g., 26), and for some skills it improved further at follow-up (e.g., 17 - moving the wheelchair down a slight incline, 31 - staying in a wheelie, moving forwards down a steep ramp). In total, approximately 60% reached a small clinically meaningful improvement (≥5 points) at completion and follow-up.

### Resilience

There were moderate effect size gains in CD-RISC total score which were not retained at the 3-month follow-up (Table 2). Regarding individual items (Supplementary Table 7), participants reported gains at programme completion on the items assessing the capacity to adapt when changes occur, to deal with whatever comes their way, to see the humorous side of things when faced with problems, and were more likely to agree that coping with stress can make one stronger. Out of these, only gains related to dealing with whatever comes my way were retained at the 3-month follow-up.

### Life satisfaction

Regarding satisfaction with life, sexual life, leisure and vocational status were the three areas where most participants were least satisfied with their life at programme start. Partner relationships, contacts with friends and acquaintances, and family life were the three areas where most participants were satisfied with their life at programme start. At an individual item level (Supplementary Table 8), more individuals were satisfied with managing self-care at the 3-month follow-up as compared to programme start (p = 0.002).

### Participation

In terms of total participation frequency and restrictions in participation scores, there were no reported gains at 3 months compared to the start of the programme. At the start of the programme, around half of the participants were engaged in sports activities at least 6–10 times per month and were going out (e.g., eating out, visiting a cafe, cinema, concert, alone or together with others) at least 3 times per month. Performing household duties was perceived as the area with most restrictions (85%), followed by engaging in work and education (74%), sports or other physical exercise (73%) and day trips (74%). At an individual item level (Supplementary Table 9), participants reported spending more time in household duties at the 3-month follow-up as compared to programme start (p = 0.024).

### Modelling change

In what follows, we report only the models in which changes in the outcome variables could be explained by at least one of the included independent variables in the multivariable model (selection threshold: p < 0.2). Of the six regression analyses conducted to model changes between start and completion of the programme, four were significant. Of the four analyses conducted to model changes between start of programme and 3-month follow-up, three were significant. All models presented in what follows exhibited no influential cases and no multicollinearity, and normally distributed residuals (i.e. no evidence of heteroscedasticity).

At completion compared to baseline, none of the independent variables included in the model could explain changes related to SCIM-SR total score, MSES total score and QEWS total score (Table 4). As for SCIM-SR domain scores, complete tetraplegia was associated with the greatest gains in independence in self-care (model adj. R^2^ = 5%) after adjusting for aetiology of injury. The shorter the time since injury the greater the gains in Personal domain of MSES (model adj. R^2^ = 5%) after adjusting for age. Female sex, being a new mentee and longer time since injury were associated with greater gains in WST capacity (model adj. R^2^ = 10%). Complete paraplegia and female sex were associated with greater gains in WST confidence (model adj. R^2^ = 13%).

**Table 4:** Multivariable regression analysis models exploring predictors of outcome gainsa.

				*Coefficients*			*Estimated marginal means*				*Model summary*	
Delta outcome	Time point comparison	Variable	Levels	B	SE	*p*-value^b^	MMD^c^	SE	Lower CI	Upper CI	Adj. R^2^	*p*-value
SCIM-SR self-care	T2–T1										0.05	0.026
		Level and completeness of SCI	Complete Para	−0.605	0.553	0.274	0.64	0.48	−0.30	1.58		
			Incomplete Para	−0.936	0.526	0.075	0.31	0.36	−0.39	1.00		
			Complete Tetra	1.129	0.559	0.043	2.37	0.49	1.42	3.32		
			Incomplete Tetra	0			1.24	0.44	0.38	2.10		
		Aetiology	Traumatic	0.516	0.569	0.365						
			Non-traumatic	0								
MSES Personal	T2–T1										0.051	0.023
		Age	increasing	−0.008	0.005	0.109						
		Years since injury	increasing	−0.034	0.013	0.011						
WST-Q capacity	T2–T1										0.154	<0.001
		Sex	Female	4.732	1.954	0.015	10.77	1.55	7.73	13.82		
			Male	0			6.04	1.20	3.70	8.39		
		Previous participation	Recurrent mentees	−5.866	1.911	0.002	5.47	1.48	2.57	8.38		
			New mentees	0			11.34	1.25	8.89	13.79		
		Years since injury	increasing years	0.452	0.165	0.006						
WST-Q confidence	T2–T1										0.131	0.001
		Sex	Female	8.763	2.854	0.002	13.67	2.29	9.18	18.16		
			Male	0			4.91	1.73	1.52	8.30		
		Level and completeness of SCI	Complete Para	9.815	4.004	0.014	17.12	2.87	11.49	22.75		
			Incomplete Para	1.050	3.765	0.780	8.36	2.50	3.46	13.25		
			Complete Tetra	−2.921	4.094	0.476	4.38	2.96	−1.42	10.19		
			Incomplete Tetra	0			7.31	2.85	1.72	12.89		
		Previous participation	Recurrent mentees	−4.224	2.873	0.141						
			New mentees	0								
SCIM-SR Total	T3–T1										0.091	0.007
		Sex	Female	2.842	0.974	0.004	3.81	0.78	2.27	5.35		
			Male	0			0.97	0.60	−0.21	2.15		
		Level and completeness of SCI	Complete Para	−0.849	1.391	0.542	2.65	1.09	0.50	4.79		
			Incomplete Para	−3.438	1.220	0.005	0.06	0.85	−1.61	1.72		
			Complete Tetra	−0.129	1.359	0.925	3.37	1.01	1.39	5.34		
			Incomplete Tetra	0			3.49	0.91	1.72	5.27		
		Previous participation	Recurrent mentees	−2.261	0.991	0.023	1.26	0.78	−0.26	2.78		
			New mentees	0			3.52	0.63	2.29	4.75		
WST-Q capacity	T3–T1										0.216	<0.001
		Sex	Female	5.686	2.036	0.005	10.33	1.60	7.21	13.46		
			Male	0			4.65	1.25	2.20	7.09		
		Previous participation	Recurrent mentees	−4.889	1.963	0.013	5.04	1.46	2.18	7.91		
			New mentees	0			9.93	1.35	7.29	12.57		
		Years since injury	increasing	0.582	0.176	<0.001						
WST-Q confidence	T3–T1										0.045	0.042
		Previous participation	Recurrent mentees	−6.973	3.312	0.035	4.17	2.44	−0.61	8.94		
			New mentees	0			11.14	2.24	6.74	15.53		

T1: start of programme (baseline); T2: programme completion; T3: 3-month follow-up.

*MMD* marginal mean difference, *SCIM-SR* Spinal Cord Independence Measure Self-Report, *MSES* Moorong Self-Efficacy Scale, *WST-Q* Wheelchair Skills Test Questionnaire.

^a^Table presents models that reach statistical significance.

^b^Bonferroni corrected.

^c^Deviation of variable level to the mean value.

At 3-month follow-up compared to baseline, being female, being a new mentee were associated with greater gains in SCIM-SR total score and incomplete paraplegia with least gains (model adj. R^2^ = 9%). Female sex, being a new mentee and longer time since injury were associated with greater gains in WST capacity (model adj. R^2^ = 22%). Being a new mentee was associated with greater gains in WST-Q confidence (model adj. R^2^ = 5%).

## Discussion

For the first time, we demonstrate that participation in short, intensive, peer-led AR programmes leads to meaningful gains in several life areas for individuals with SCI. We found improvements in self-reported physical independence, and specifically in dressing and washing, bowel management, bed mobility and transfers for those in need of practicing these tasks. Both self-reported and objectively measured wheelchair skills showed sustained improvements. Improvements in aspects of self-efficacy and resilience seen at programme completion were not present at the 3-month follow-up. With few exceptions, the outcome predictors examined in this study did not account for the observed outcome gains.

The AR approach is distinct to other peer mentorship programmes in several ways. AR offers time-limited residential programmes that are long enough to allow mentees to connect with peer mentors, “build a temporary community”, and engage in both physical and mental training [19]. These programmes require participants to leave their everyday environments and support systems, encouraging them to step outside their comfort zones [19]. Our fidelity criteria demonstrate that the programmes include a demanding schedule of structured training sessions, as well as ample opportunities for informal interaction with peer mentors and fellow mentees—during breaks, rest periods, and in shared accommodations. Trained peer mentors deliver nearly all scheduled sessions and have a high ratio to mentees. This increases the likelihood that each mentee will find at least one mentor they can relate to and connect with. Our previous research clearly indicates that peer mentors take pride in their roles, feel personally rewarded by supporting others in similar situations, and find the programmes beneficial for their personal growth [19]. Although the current study cannot determine which specific components contribute most to the observed positive outcomes, it is likely that the combination of these factors helps create an enabling learning environment [6, 17].

### Reported complications

Among the 111 participants across the eight training programmes, there was one reported fall resulting in a fracture, sprain or similar injury, and five additional mentees reported skin injuries due to falls or other activities. Severe adverse events, such as fractures, are generally uncommon in activity-based rehabilitation programmes, while non-severe events—such as skin abrasions and pain—are more frequently observed in people with SCI [33]. In our study, uncomplicated falls were common, suggesting that mentees frequently pushed beyond their comfort zones to practice skills they had not yet mastered. At three months follow-up, fewer individuals reported serious falls and fear with performing activities. Previous research has shown that individuals with SCI see peer training, especially in a group setting, as the preferred option for fall prevention and fall management training [34] The degree that increased mastery during the AR camps is related to reduction in complicated falls, risk of falling and fear of performing activities needs to be investigated further. Overall, despite their short duration and high intensity, the peer-led AR training programmes appear safe, with a low risk of serious injury.

### Physical independence

Gains in physical independence were substantial, with one in three mentees achieving at least small clinically meaningful improvements both at programme completion and at the 3-month follow-up. The largest changes during the programme were reported in the self-care domain (SCIM-SR), which were further supported by an increased proportion of mentees reporting satisfaction with managing self-care (LiSat-11) compared to programme start. Self-care and mobility are key targets during hospital-based rehabilitation. However, during that phase of rehabilitation, some individuals may not have reached their full rehabilitation potential [17]. Further, some of the skills acquired are often lost during the transition to home environment due to the “multidimensional change of context” from hospital to home [1]. Previous research has emphasised the need for specialised transition teams to bridge the gap between hospital and home [1].

In AR training programmes, the acquisition context–community-based environment–more closely resembles the real-world application context of the home environment, which we believe facilitates both skill acquisition and transfer. Mentees practice self-care and mobility tasks intensively, supported by peer mentors through a combination of formal sessions (explicit learning) and informal interactions (implicit learning). For example, by sharing room, mentees observe how mentors perform daily activities in a natural setting, modelling strategies to meet the demanding pace of the training programme. In this context, our results indicate that peer-led AR programmes can serve as a valuable resource to further advance and retain important skills during the critical transitional phase following discharge.

Mentees achieved gains in bowel-related items across outcome measures. More specifically, participants reported gains at the 3-month follow-up in managing their bowel movements (SCIM-SR), in being more confident with avoiding bowel accidents (MSES), wheelchair to toilet transfer, and toilet use (SCIM-SR). Toilet use and bowel management were two areas where the vast majority of participants were below the highest possible score at the start of the programme, suggesting possible large unmet needs. Given the difficulty of the task toilet use and the lack of improvement at follow-up, future programmes might benefit from focusing more on how to facilitate the retention of this specific task in the home environment. Bowel dysfunction is common in people with SCI [35] and bowel management is a sensitive and intimate area where people with SCI may not feel comfortable discussing openly after discharge from rehabilitation. Our results support the notion that peer mentorship can be a prominent facilitator to rapidly changing bowel care practices [35], which in turn can lead to improved bowel management and function entailing positive effects on quality of life [36].

In a Day program (median duration of 17 days) led by therapists, 114 individuals (median age 25 years; median time since injury 3 months) achieved gains across all SCIM-III domains [37]. In contrast, another intervention study that provided a therapist-led, activity-based programme three times per week for 12 weeks (median age 40–43 year; median time since injury 4–6 years), found no effects on SCIM scores at programme completion or at the 6-month follow-up [33]. In our study, the programme was shorter (7 days), more intensive, and led by peer mentors. Participants had a median age of 43 years, and a median time since injury of 1 year. Taken together, these comparisons suggest that factors such as time since injury, intervention intensity, and the background of the person delivering the intervention may influence the effectiveness of training programmes for community dwelling individuals with SCI.

### Self-efficacy, resilience and participation

There were improvements in all domains related to self-efficacy at programme completion, where half of participants achieved at least a small clinically meaningful gain. Improvements were found in relation to confidence in finding hobbies and leisure, having a fulfilling lifestyle, and being an active member of the household. Further, participants reported feeling more confident in dealing with unexpected problems (MSES; CD-RISC) both at completion and follow-up. Overall, self-efficacy and resilience scores were improved at completion, but not at follow-up. Previous research shows that the interaction with peer mentors helps participants with SCI to explore their “unrealised potential” [17]. In other words, that interacting with peers who have found hobbies, who are active members of a household and can be considered to have fulfilling lifestyles instils confidence in mentees about their own future and what they are able to achieve despite the injury.

Frequency in household duties was the single area where mentees made improvements in participation. This improvement in USER-P is consistent with large improvements in self-efficacy related to family life and specifically to being an active member of the household and maintaining contact with people. The limited effects in other aspects of community participation were not a surprise given the short follow-up time of the present study. Higher disability management self-efficacy has been found to positively correlate with higher participation [38]. Based on that, while the present study did not find any other improvements in community participation, the observed changes in confidence related to participation in valued situations and activities may be a facilitator for improving aspects of participation in a longer perspective. Future research should further explore effects on community participation and contributing mechanisms with longer follow-up times.

### Wheelchair skills

Sixty percent or more of mentees reached at least small clinically meaningful changes in both capacity and confidence in wheelchair skills, measured both objectively and by self-report. Positive gains were seen both at completion and follow-up. Wheelchair skills training is recommended during initial rehabilitation, and has been associated with return to work and higher community participation [39]. However, it may be deprioritised in favour of other important life areas during initial rehabilitation, and patients are unlikely to receive the wheelchair they will use in everyday life during this phase [34]. Arenas for practicing wheelchair skills, other than in-patient rehabilitation, are therefore important. Our results highlight the effectiveness of intensive, residential, peer-led programmes to improve wheelchair skills after return to community life after SCI.

### Predictors

Very few of the independent variables could explain outcomes. Our results indicate that females may benefit even more from AR training programmes regarding improving self-reported wheelchair skills at both completion and after 3 months, and physical independence after 3 months. In addition, our results indicate that the largest improvements in these aspects take place during the first or second AR training programme. However, the independent variables explained only little variance in the outcomes. Further studies could focus on modifiable factors associated with improvements during and after AR training programmes. The lack of predictors known to influence gains during initial in-patient rehabilitation [40] is not surprising in this self-selected sample attending secondary community-based rehabilitation. We assume that participants of AR programmes had already achieved gains during the initial in-patient and out-patient rehabilitation. In that way, our findings show the added value of community rehabilitation programmes, as a complement to completed comprehensive hospital-based rehabilitation.

In total, 12% of participants in the current study were older than 60 years of age. Given the increase in mean age at injury during the last 20 years in Nordic countries [20], this study cohort of mentees participating in AR programmes differs from the incident cohort with SCI in Sweden. Further, 40% of our cohort had lived with SCI for more than two years. Our findings show that neither age nor time since injury were predictors for outcome gains. This indicates that both older people and those living with SCI for a longer time have good chances of benefitting from participating in AR programmes.

### Temporality of gains

Gains achieved during the intensive AR residential programmes were substantial, with 39% achieving small clinically meaningful improvements in SCIM-SR at programme completion and 34% at the 3-month follow-up. In some areas, especially related to self-efficacy and resilience, participants reported improvements at programme completion which were not maintained over time. These results suggest a “booster effect”, where mentees temporarily experience elevated self-efficacy and resilience, likely related to interactions with peer mentors and peers at the supportive environment of AR programmes. Although these perceived gains may diminish over time, their short-term impact may be critical–especially for individuals who struggle to initiate or sustain behavioural change.

According to Bandura’s self-efficacy theory, performance accomplishments are the most powerful source of self-belief, as they provide direct evidence of capability [41]. Even brief successful experiences can influence future coping behaviour [41], enabling individuals to recall and draw upon the experience long after the behaviour itself has lapsed [41]. This is particularly relevant for individuals with low baseline self-efficacy, who often avoid situations they perceive as exceeding their coping ability. Gaining “corrective experiences” in a supportive setting can break this cycle and “mobilize greater effort” in future challenges [41]. Additionally, “occasional failures that are later overcome by determined effort can strengthen self-motivated persistence”, suggesting that early, even fleeting, gains may support future re-engagement [41]. Future research should explore how the timing and sequencing of AR programmes and healthcare rehabilitation can be designed to reinforce and consolidate these early mastery experiences, in order to achieve larger and more durable behavioural change for people with SCI.

### Methodological considerations–strengths and limitations

While the current manuscript is in line with the published INTER-PEER protocol [13], we are hereby presenting additional analyses, e.g., the computation of minimal clinically important difference, analysis of effects at an item level and the regression analysis. The need for this type of analysis emerged during the study and after we published the protocol, therefore it is not presented in the protocol.

AR programmes last on average 7 days in Sweden. Therefore, a small clinically meaningful change should be considered a key outcome of interest. Interestingly, the thresholds for SCIM-SR from the current study are very similar to those reported in relation to SCIM-III [29], supporting the suitability of our analysis. We believe that such analyses should be considered in the future design of controlled studies exploring the effectiveness of AR training programmes.

We have used total and domain scores for ordinally measured variables where it cannot be assumed that scores are equidistant. We do, however, have confidence in our findings as we also investigated changes at the item level. Moreover, analysing total and domain scores is common procedure for many of these outcome measures [42–44]. In some instances (e.g. self-care domain score at follow-up), despite a lack of significant changes at a domain level, substantial improvements could be observed in several individual items. This supports the importance of looking into individual tasks and not just domain scores when analysing effects on physical independence.

Our response and retention rates were very good, and there was little missing data. It can be argued that the cohort of individuals participating in AR training programmes may be highly motivated, and therefore not representative of the general SCI population. Also, participation is mainly through self-referral.

Due to the long time since injury, it is unlikely that the observed improvements were spontaneous. Although we did not monitor if participants conducted any other interventions between completion and the 3-month follow-up, it is unlikely that such interventions could have influenced our outcomes because of the rather short follow-up time.

Two of the co-authors in this study have a lived experience of SCI. Both have been involved and provided input in all stages of the project, which we believe has strengthened the interpretation of study findings.

Last, but not least, the reader should interpret our findings with caution, as INTER-PEER is an observational study that relies predominantly on self-reported measures. At the same time, this study represents an important step forward in establishing the effects of peer-led AR training programmes and provides a strong platform for future intervention research. Moreover, while the current study addresses the “what” in terms of gains, answering the “how” questions–such as the mechanisms behind these effects–will require qualitative research approaches.

## Conclusion

This is the first comprehensive, scientific evaluation of the effects of peer-led, community AR training programmes on mentees with SCI in the Swedish context. AR training programmes should be considered as an effective and low-cost opportunity to improve important and challenging aspects of physical independence and wheelchair skills after discharge from in-hospital rehabilitation. The AR programmes are also effective in improving aspects of self-efficacy and resilience. Although some gains were not sustained at follow-up, we argue that the short-term improvements observed at programme completion may serve as critical mastery experiences that enhance long-term behavioural change, particularly for individuals who struggle to initiate or maintain progress. These findings strongly advocate that intensive residential peer-led programmes taking place in the community, such as AR, play a crucial role in the rehabilitation continuum for people with SCI. Findings from the current study support the systematic implementation of such programmes as a complement to clinical rehabilitation processes, and making them readily available to more individuals with SCI.

## Supplementary information

Supplemental material

## Data Availability

Aggregated data are available upon reasonable requests to the principal investigator.
