# Supplementary material for: Effects of community peer-led programmes for people with spinal cord injury in Sweden–the INTERnational project for the evaluation of active rehabilitation (INTER-PEER)
Source: Spinal Cord. 2025 Oct 6;63(11):567–78. doi: 10.1038/s41393-025-01119-4 (PMC12583193; doi:10.1038/s41393-025-01119-4)
Supplement: Supplementary file 1 — Supplemental material [file 41393_2025_1119_MOESM1_ESM.pdf]

Supplementary Information

Effects of community peer-led programmes for people with spinal cord injury in Sweden – the INTERnational Project for the Evaluation of Active Rehabilitation (INTER-PEER)

Anestis Divanoglou, PhD[1]; Erik Berndtsson[2]; Tomasz Tasiemski, PhD[3]; Carolina Saskia Fellinghauer, PhD[4]; & Sophie Jörgensen, PhD[5]

Table of contents

Supplementary Table 1. Thresholds for small clinically meaningful change in primary INTER-PEER outcome measures.

Supplementary Table 2. Fidelity criteria for 8 consecutive Active Rehabilitation training programmes in Sweden.

Supplementary Table 3. Reported complications prior, during and after the AR training programme in Sweden.

Supplementary Table 4. Changes in individual items of SCIM-SR comparing the commencement (T1, baseline) with the completion of the training programme (T2), and with 3 months after the end of the AR programme (T3).

Supplementary Table 5. Changes in individual items of MSES comparing the commencement (T1, baseline) with the completion of the training programme (T2), and with 3 months after the end of the AR programme (T3).

Supplementary Table 6a. Changes in individual items of WST capacity comparing the commencement (T1, baseline) with the completion of the training programme (T2), and with 3 months after the end of the AR programme (T3).

Supplementary Table 6b. Changes in individual items of WST confidence comparing the commencement (T1, baseline) with the completion of the training programme (T2), and with 3 months after the end of the AR programme (T3).

Supplementary Table 7. Changes in particular items of CD-RISC comparing the commencement (T1, baseline) with the completion of the training programme (T2), and with 3 months after the end of the AR programme (T3).

Supplementary Table 8. Changes in particular items of LiSat-11 over two timepoints of measurement (T1, T3).

Supplementary Table 9. Changes in individual items of USER-Participation over two timepoints of measurement (T1, T3).

Supplementary Table 1. Thresholds for small clinically meaningful change in primary INTER-PEER outcome measures.

**Outcome measures and respective domains**	**SD T1**	**Thresholds for clinically important difference – small**
MSES General	1,1	0.2
MSES Personal	1,3	0.3
MSES Social	1,1	0.2
MSES Total	1,0	0.2
QEWS	5,0	1.0
SCIM 1-4	4,9	1.0
SCIM 5-8	7,6	1.0
SCIM 9-11	2,9	1.0
SCIM 12-17	7,1	1.0
SCIM Total	18,6	4.0
WSTQ capacity	23,0	5.0
WSTQ confidence	24,7	5.0
Thresholds for small clinically important differences were determined as significant if the change was larger than SDstart x 0.2 [27, 28]		

SD: standard deviation; T1: programme start; MSES: Mooorong Self-efficacy Scale; QEWS: Queensland Evaluation of Wheelchair Skills; SCIM: Spinal Cord Independence Measure Self-report; WST-Q: Wheelchair Skills test Questionnaire

Supplementary Table 2. Fidelity criteria for 8 consecutive Active Rehabilitation training programmes in Sweden.

	MEAN	MIN	MAX	SD	Proportion of programs meeting criterion
1a) Peer mentors with a ratio of at least one peer mentor for every five participants; (ratio peer mentors:participants)		1:2	1:1		8/8
1b) Peer mentors leading a minimum of 80% of all structured sessions; (% of peer mentors)	100	96	100	0	8/8
2) Trained non-disabled assistants;					8/8
3) ADL and wheelchair skills training provided by peer mentors on average at least 90 minutes daily; (minutes)	105	69	145	30	5/8
4) Physical training. sports and therapeutic recreation activities incorporated in the schedule on average at least 90 minutes daily; (minutes)	110	69	175	32	6/8
5) Formal educational sessions (e.g. on bowel and bladder management; sexual function) on average at least 30 minutes daily; (minutes)	65	43	90	14	8/8
6) Training environment in the community;					8/8
7) Specific admission criteria (i.e. being free of severe medical complications; being able to push a manual wheelchair on an even surface; being able to follow instructions; not having problems with concentration, memory and orientation);					8/8
- At least half of the participants having a SCI; (n)					8/8
- Participants with a SCI; (n)	14	9	24	5	
- At least half of the peer mentors having a SCI; (n)					8/8
- Peer mentors with a SCI; (n)	11	7	18	4	8/8
8) A goal-setting process is part of the programme;					8/8
9) Peer mentors having received formal training about AR;					8/8
10) Duration of the AR programmes between 7-15 days; (days)	8	7	11	2	8/8
- Included participants attending at least 3/4 of the actual programme duration; (n)					8/8

Supplementary Table 3. Reported complications prior, during and after the AR training programme in Sweden

Reported complications (n, %)[a]	Complications during last 3 months prior to programme start<br>65 (59%)	Complications during the programme (7 days)<br>50 (45%)	Complications during the last 3 months after programme completion<br>75 (68%)
Fall that resulted in a fracture, sprain or sth similar	4 (4%)	1 (1%)	1 (1%)
Skin injury due to fall or other activity	9 (8%)	5 (5%)	8 (7%)
Fall that did not result in an injury	26 (23%)	31 (28%)	37 (33%)
More frequently than usual urinary tract infection	26 (23%)	3 (3%)	18 (16%)
Prolonged fatigue	20 (18%)	15 (14%)	22 (20%)
Pressure ulcer	6 (5%)	0 (0%)	8 (7%)
Emotional breakdown	22 (20%)	12 (11%)	23 (21%)
Increased fear with performing activities	13 (12%)	1 (1%)	7 (6%)
Increased concern for the future in relation to SCI	31 (28%)	7 (6%)	35 (32%)

[a]Percentages are calculated based on total number of participants (N=111)

Supplementary Table 4. Changes in individual items of SCIM-SR comparing the commencement (T1, baseline) with the completion of the training programme (T2), and with 3 months after the end of the AR programme (T3)

MSES item	Domain	T1	T2 vs T1			T3 vs T1		
		**N valid (% Below highest possible score)**	**N**	**Improved, n (%) of those below highest possible score at T1**	**P value[a]**	**N**	**Improved, n (%) of those below highest possible score at T1**	**P value[a]**
1. Eat	Self-care	111 (25%)	111	8 (29%)	0.058	108	6 (21%)	0.317
2.a. Wash UB	Self-care	111 (69%)	111	29 (38%)	**<.001**	111	22 (29%)	**0.002**
2.b. Wash LB	Self-care	111 (79%)	111	27 (31%)	**<.001**	111	21 (24%)	**0.002**
3.a. Dress UB	Self-care	111 (32%)	111	21 (58%)	**<.001**	111	15 (42%)	**0.011**
3.b. Dress LB	Self-care	111 (46%)	111	28 (55%)	**<.001**	111	26 (51%)	**0.034**
4. Grooming	Self-care	111 (19%)	111	13 (62%)	**0.040**	108	11 (52%)	**0.022**
5. Breathing	Respiration & sphincter	111 (14%)	111	6 (40%)	0.058	108	8 (53%)	0.396
6. Bladder	Respiration & sphincter	110 (89%)	110	14 (14%)	0.055	106	15 (15%)	0.691
7. Bowel	Respiration & sphincter	110 (75%)	109	18 (23%)	0.335	105	20 (25%)	**0.023**
8. Toilet use	Respiration & sphincter	111 (93%)	111	22 (21%)	**0.003**	108	15 (15%)	0.070
9. Mobility in bed & in w/c	Mobility in room and toilet	111 (32%)	111	15 (41%)	0.161	108	16 (43%)	**0.001**
10. Bed to w/c transfer	Mobility in room and toilet	111 (43%)	111	10 (21%)	**0.021**	108	10 (21%)	0.109
11. w/c to Toilet transfer	Mobility in room and toilet	111 (60%)	111	18 (28%)	**0.050**	108	23 (36%)	**0.007**
12. Moving indoors	Mobility indoors and outdoors on even surfaces	111 (91%)	111	6 (35%)	0.694	108	7 (41%)	0.076

13. Moving 10-100m	Mobility indoors and outdoors on even surfaces	111 (94%)	111	10 (40%)	0.550	108	11 (44%)	0.722
14. Moving >100m	Mobility indoors and outdoors on even surfaces	111 (96%)	111	9 (27%)	0.808	107	9 (24%)	0.846
15. Walk stairs	Mobility indoors and outdoors on even surfaces	111 (87%)	111	13 (14%)	0.414	109	13 (14%)	0.253
16. w/c to car transfer	Mobility indoors and outdoors on even surfaces	111 (60%)	111	23 (35%)	**<.001**	108	19 (29%)	0.657
17. Floor to w/c transfer	Mobility indoors and outdoors on even surfaces	111 (71%)	111	13 (16%)	**0.001**	108	11 (14%)	**0.004**

Note
[a]Wilcoxon non-parametric signed-rank test to identify score changes between respective time points
w/c: wheelchair; UB: upper body; LB: lower body; T1: programme start; T2: programme completion; T3: 3 months follow-up

Supplementary Table 5. Changes in individual items of MSES comparing the commencement (T1, baseline) with the completion of the training programme (T2), and with 3 months after the end of the AR programme (T3)

MSES item	Domain	**T1**	**T2 vs T1**			**T3 vs T1**		
		**N valid (% Below highest possible score)**	**N**	**Improved, n (%) of those below highest possible score at T1**	**P value[a]**	**N**	**Improved, n (%) of those below highest possible score at T1**	**P value[a]**
**1.** Maintain my personal hygiene	Personal	111 (53%)	111	29 (49%)	0.150	107	30 (51%)	0.254
**2.** Avoid bowel accidents	Personal	111 (78%)	111	42 (48%)	**<.001**	107	39 (45%)	**0.014**
3. Active member of the household	Personal	110 (71%)	110	38 (49%)	**<.001**	106	34 (44%)	0.276
4. Maintain relations in my family	Social	111 (47%)	111	21 (40%)	0.261	107	19 (37%)	0.691
5. Get out of my house	Personal	110 (66%)	110	34 (47%)	**0.019**	107	27 (38%)	0.669
6. Satisfying sexual relation	Other	108 (89%)	107	34 (36%)	**0.021**	103	32 (34%)	0.464
7. Spending time with my friends	Social	110 (49%)	110	26 (48%)	0.121	106	24 (44%)	0.609
8. Find hobbies and leisure	Social	111 (73%)	109	41 (52%)	**<.001**	107	33 (42%)	0.444
9. Maintain contact with people	Social	111 (59%)	110	30 (65%)	**0.012**	106	26 (57%)	0.538
10. Deal with unexpected problems	General	111 (81%)	110	38 (48%)	**<.001**	106	39 (49%)	0.078
11. Being able to work	Social	109 (54%)	108	29 (50%)	0.164	104	21 (36%)	0.305
12. Accomplish most things	General	110 (76%)	110	23 (27%)	**0.023**	104	29 (35%)	0.913
13. Learn something new	General	111 (71%)	110	23 (29%)	0.598	106	26 (33%)	0.585
14. Make the first contact	General	110 (77%)	109	36 (43%)	**0.021**	106	31 (37%)	0.703
15. Maintain good health and well-being	Other	108 (81%)	108	31 (36%)	0.061	106	31 (36%)	0.144

16. Having a fulfilling lifestyle in the future	Other	111 (75%)	110	36 (44%)	**0.001**	106	25 (30%)	0.829

Note

[a]Wilcoxon non-parametric signed-rank test to identify score changes between respective time points

MSES: Moorong Self-efficacy Scale; w/c: wheelchair; UB: upper body; LB: lower body; T1: programme start; T2: programme completion; T3: 3 months follow-up

Supplementary Table 6a. Changes in individual items of WST capacity comparing the commencement (T1, baseline) with the completion of the training programme (T2), and with 3 months after the end of the AR programme (T3)

		T1	T2 vs T1			T3 vs T1		
**WST-Q item - capacity**	**Level of difficulty**	**N valid (% Below highest possible score)**	**N**	**Improved, n (%) of those below highest possible score at T1**	**P value[a]**	**N**	**Improved, n (%) of those below highest possible score at T1**	**P value[a]**
**10. Removing the weight from your buttocks**	B	103 (24%)	101	9 (36%)	0.827	94	10 (40%)	**0.021**
**12. Folding your wheelchair or taking it apart without tools**	I	101 (58%)	100	15 (25%)	**0.012**	89	12 (20%)	**0.039**
**13. Opening a door, moving through it and closing it behind you**	I	101 (37%)	99	19 (51%)	**0.002**	91	16 (43%)	**0.050**
**14. Moving the wheelchair over a longer distance**	I	102 (15%)	101	8 (53%)	**0.020**	94	9 (60%)	0.166
**15. While moving the wheelchair, avoiding moving people**	I	101 (10%)	100	7 (70%)	0.646	93	7 (70)	0.207
**16. Moving the wheelchair up a slight incline**	I	101 (36%)	101	21 (58%)	**0.004**	74	13 (36%)	**0.050**
**17. Moving the wheelchair down a slight incline**	I	102 (21%)	100	10 (48%)	0.073	94	8 (38%)	0.808
**18. Moving the wheelchair up a steep incline**	A	103 (69%)	103	25 (35%)	**0.001**	96	25 (35%)	**0.012**
**19. Moving the wheelchair down a steep incline**	A	101 (51%)	101	22 (43%)	0.086	94	23 (45%)	0.064
**20. Moving the wheelchair across a slight side-slope**	I	102 (48%)	102	23 (47%)	**0.002**	95	22 (45%)	**0.002**
**21. Moving the wheelchair a short distance across a soft surface**	I	102 (48%)	102	28 (57%)	**<0.001**	95	23 (47%)	**0.001**
**22. Getting the wheelchair over an obstacle above the surface**	I	102 (18%)	102	9 (50%)	0.134	94	8 (44%)	0.058
**23. Getting the wheelchair over a gap**	I	101 (53%)	100	35 (66%)	**<0.001**	95	24 (45%)	**0.002**
**24. Getting the wheelchair up a low curb**	I	101 (35%)	101	18 (51%)	0.065	95	17 (49%)	0.562
**25. Getting the wheelchair down from a low curb**	I	101 (32%)	101	20 (63%)	**0.023**	94	17 (53%)	**0.038**
**26. Getting the wheelchair up a high curb**	A	101 (82%)	100	33 (40%)	**<0.001**	93	34 (41%)	**<0.001**
**27. Getting the wheelchair down from a high curb**	A	101 (68%)	101	35 (51%)	**<0.001**	93	23 (33%)	**<0.001**

**28. Doing a wheelie for 30 seconds**	A	100 (37%)	100	23 (62%)	**<0.001**	93	20 (54%)	**0.001**
**29. Staying in a wheelie, turning the wheelchair around**	A	101 (55%)	101	29 (52%)	**<0.001**	94	30 (57%)	**<0.001**
**30. Staying in a wheelie, moving forwards down a high curb**	A	101 (73%)	101	26 (35%)	**<0.001**	94	26 (35%)	**<0.001**
**31. Staying in a wheelie, moving forwards down a steep ramp**	A	101 (75%)	100	28 (37%)	**<0.001**	94	26 (34%)	**<0.001**
**33. Getting yourself and the wheelchair up a short flight of stairs that has a rail**	A	25 (92%)	24	7 (30%)	**0.015**	21	7 (30%)	**<0.001**
**34. Getting yourself and the wheelchair down a short flight of stairs that has a rail**	A	25 (84%)	24	9 (43%)	**0.006**	11	4 (19%)	**0.046**

Note

[a]Wilcoxon non-parametric signed-rank test to identify score changes between respective timepoints

w/c: wheelchair; UB: upper body; LB: lower body; B: Basic level of difficulty; I: Intermediate level of difficulty; A: Advanced level of difficulty

Supplementary Table 6b. Changes in individual items of WST confidence comparing the commencement (T1, baseline) with the completion of the training programme (T2), and with 3 months after the end of the AR programme (T3)

		T1	T2 vs T1			T3 vs T1		
**WST-Q items - confidence**	**Level of difficulty**	**N valid (% Below highest possible score)**	**N**	**Improved, n (% improved/ below highest possible score)**	**P value***	**N**	**Improved, n (% improved/ below highest possible score)**	**P value***
**10. Removing the weight from your buttocks**	B	97 (34%)	93	15 (45%)	**0.047**	67	10 (30%)	0.197
**12. Folding your wheelchair or taking it apart without tools**	I	91 (58%)	86	16 (37%)	0.120	63	14 (33%)	**0.021**
**13. Opening a door, moving through it and closing it behind you**	I	97 (50%)	93	23 (48%)	**0.005**	69	16 (33%)	**0.028**
**14. Moving the wheelchair over a longer distance**	I	97 (31%)	94	17 (57%)	**0.002**	69	14 (47%)	**0.005**
**15. While moving the wheelchair, avoiding moving people**	I	96 (43%)	94	15 (37%)	0.317	68	13 (32%)	0.108
**16. Moving the wheelchair up a slight incline**	I	95 (50%)	93	22 (47%)	**0.024**	66	16 (34%)	**0.014**
**17. Moving the wheelchair down a slight incline**	I	96 (42%)	96	23 (58%)	**0.004**	91	42 (100%)	**<.001**
**18. Moving the wheelchair up a steep incline**	A	93 (71%)	91	20 (30%)	0.286	77	18 (27%)	0.097
**19. Moving the wheelchair down a steep incline**	A	94 (70%)	92	26 (39%)	0.120	79	22 (33%)	0.133
**20. Moving the wheelchair across a slight side-slope**	I	97 (73%)	95	23 (32%)	**0.009**	83	26 (37%)	**0.002**
**21. Moving the wheelchair a short distance across a soft surface**	I	96 (56%)	94	27 (50%)	**0.006**	82	19 (35%)	**0.034**
**22. Getting the wheelchair over an obstacle above the surface**	I	98 (39%)	96	21 (55%)	**0.046**	84	19 (50%)	0.057
**23. Getting the wheelchair over a gap**	I	95 (74%)	92	35 (50%)	**<.001**	82	30 (33%)	**<.001**
**24. Getting the wheelchair up a low curb**	I	97 (67%)	95	26 (40%)	**<.001**	82	26 (40%)	**0.002**
**25. Getting the wheelchair down from a low curb**	I	96 (59%)	94	27 (47%)	**<.001**	82	24 (42%)	**0.001**
**26. Getting the wheelchair up a high curb**	A	92 (86%)	88	21 (27%)	**0.038**	75	21 (27%)	**0.017**

**27. Getting the wheelchair down from a high curb**	A	93 (75%)	91	32 (46%)	**<.001**	76	20 (29%)	**0.005**
**28. Doing a wheelie for 30 seconds**	A	94 (46%)	91	22 (51%)	0.107	80	19 (44%)	**0.041**
**29. Staying in a wheelie, turning the wheelchair around**	A	92 (72%)	90	33 (50%)	**0.002**	78	26 (39%)	**<.001**
**30. Staying in a wheelie, moving forwards down a high curb**	A	91 (80%)	88	28 (38%)	**0.002**	77	19 (26%)	**0.006**
**31. Staying in a wheelie, moving forwards down a steep ramp**	A	90 (82%)	87	24 (32%)	**0.003**	84	38 (51%)	**<.001**
**33. Getting yourself and the wheelchair up a short flight of stairs that has a rail**	A	22 (86%)	20	10 (53%)	**0.022**	18	9 (47%)	**0.006**
**34. Getting yourself and the wheelchair down a short flight of stairs that has a rail**	A	22 (77%)	21	11 (65%)	**0.017**	19	9 (53%)	**0.013**

Note
*Wilcoxon non-parametric signed-rank test to identify score changes between respective timepoints
w/c: wheelchair; UB: upper body; LB: lower body; B: Basic level of difficulty; I: Intermediate level of difficulty; A: Advanced level of difficulty

Supplementary Table 7. Changes in particular items of CD-RISC comparing the commencement (T1, baseline) with the completion of the training programme (T2), and with 3 months after the end of the AR programme (T3)

CD-RISC ITEM	**T1**	**T2 VS T1**			**T3 VS T1**		
	**N valid (% Below highest possible score)**	**N**	**Improved, n (%) of those below highest possible score at T1**	**P value**	**N**	**Improved, n (%) of those below highest possible score at T1**	**P value**
1. I am able to adapt when changes occur.	74 (78%)	73	23 (32%)	**0.001**	68	16 (24%)	0.083
2. I can deal with whatever comes my way.	73 (84%)	71	24 (34%)	**0.009**	67	22 (33%)	**0.006**
3. I try to see the humorous side of things when I am faced with problems.	73 (74%)	72	21 (29%)	**0.049**	67	15 (22%)	0.992
4. Having to cope with stress can make me stronger.	74 (87%)	73	23 (32%)	**0.003**	67	14 (21%)	0.739
5. I tend to bounce back after illness, injury, or other hardships.	73 (59%)	72	20 (28%)	0.064	68	17 (25%)	0.866
6. I believe I can achieve my goals, even if there are obstacles.	74 (74%)	72	21 (29%)	0.077	68	19 (28%)	0.247
7. Under pressure, I stay focused and think clearly.	74 (88%)	71	17 (24%)	0.061	68	21 (31%)	0.088
8. I am not easily discouraged by failure.	73 (81%)	72	16 (22%)	0.346	66	19 (29%)	0.934
9. I think of myself as a strong person when dealing with life's challenges and difficulties.	73 (66%)	72	19 (26%)	0.077	67	18 (27%)	0.895
10. I am able to handle unpleasant or painful feelings like sadness, fear, and anger.	74 (80%)	73	19 (26%)	0.071	68	17 (25%)	0.342

Note

Wilcoxon non-parametric signed-rank test

Supplementary Table 8. Changes in particular items of LiSat-11 over two timepoints of measurement (T1, T3)

LiSat item	N	Satisfied T1 n (%)	Satisfied T3 n (%)	*p-value*[a]
**1.** Life as a whole	103	43 (42%)	45 (44%)	0.885
**2.** Vocational status	102	35 (34)	33 (32%)	0.824
**3.** Financial situation	104	47 (45%)	48 (46%)	1.000
**4.** Leisure	104	33 (32%)	44 (42%)	0.071
**5.** Contacts with friends and acquaintances	103	64 (62%)	66 (64%)	0.839
**6.** Sexual life	100	17 (17%)	15 (15%)	0.774
**7.** Manage Self-care	102	46 (45%)	62 (61%)	**0.002**
**8.** Family life	78	52 (67%)	58 (74%)	0.263
**9.** Partnership relation	61	37 (61%)	35 (57%)	0.791
**10.** Physical health	102	42 (41%)	46 (45%)	0.627
**11.** Psychological health	94	47 (50%)	48 (51%)	1.000

Note
[a] McNemar test
Satisfied: scores 5-7

Supplementary Table 9. Changes in individual items of USER-Participation over two timepoints of measurement (T1, T3)

ITEM				
**FREQUENCY**[B]	**N**[a]	**Improved T3_T1**		***p-value***
1. Paid work (hours)	104	10		0.437
2. Unpaid work (hours)	104	13		0.956
3. Education (hours)	103	9		0.881
4. Household duties (hours)	105	32		**0.024**
5. Sports	106	35		0.979
6. Going out	105	30		0.512
7. Day trips	104	24		0.287
8. Leisure activities at home	106	28		0.538
9. Visiting family or friends	96	24		0.438
10. Being visited by family or friends	103	30		0.797
11. Contacting others	106	31		0.660
**RESTRICTIONS**[C]	**N**[a]	**Restricted T1**	**Restricted T3**	***p-value***
12. Work or education	53	39 (74%)	35 (66%)	0.424
13. Household duties	101	86 (85%)	81 (80%)	0.359
14. Outdoor mobility	99	66 (67%)	61 (62%)	0.424
15. Sports	89	64 (72%)	64 (72%)	1.000
16. Going out	97	59 (61%)	58 (60%)	1.000
17. Day trips	99	73 (74%)	67 (68%)	0.286
18. Leisure activities at home	87	47 (54%)	37 (43%)	0.052
19. Partner relationship	60	43 (72%)	35 (58%)	0.057
20. Visiting family or friends	102	65 (64%)	65 (64%)	1.000
21. Being visited by family or friends	104	22 (21%)	25 (24%)	0.664
22. Contacting others	105	9 (1%)	4 (4%)	0.227

Note

[a]*Only cases with a relevant and valid answer are included in the analysis*

[b]Wilcoxon non-parametric signed-rank test

[c]McNemar test
